# An immunologically friendly classification of non-peptidic ligands

**DOI:** 10.1093/database/baab014

**Published:** 2021-03-27

**Authors:** Lindy Edwards, Rebecca Jackson, James A Overton, Randi Vita, Nina Blazeska, Bjoern Peters, Alessandro Sette

**Affiliations:** Center for Infectious Disease and Vaccine Research, La Jolla Institute for Immunology, 9420 Athena Circle La Jolla, CA 92037, USA; Center for Infectious Disease and Vaccine Research, La Jolla Institute for Immunology, 9420 Athena Circle La Jolla, CA 92037, USA; Knocean Inc., 107 Quebec Ave. Toronto, Ontario, M6P 2T3, Canada; Center for Infectious Disease and Vaccine Research, La Jolla Institute for Immunology, 9420 Athena Circle La Jolla, CA 92037, USA; Knocean Inc., 107 Quebec Ave. Toronto, Ontario, M6P 2T3, Canada; Center for Infectious Disease and Vaccine Research, La Jolla Institute for Immunology, 9420 Athena Circle La Jolla, CA 92037, USA; Center for Infectious Disease and Vaccine Research, La Jolla Institute for Immunology, 9420 Athena Circle La Jolla, CA 92037, USA; Center for Infectious Disease and Vaccine Research, La Jolla Institute for Immunology, 9420 Athena Circle La Jolla, CA 92037, USA; Department of Medicine, Division of Infectious Diseases and Global Public Health, University of California, 9500 Gilman Drive MC 0507 La Jolla, CA 92093-0507, USA; Center for Infectious Disease and Vaccine Research, La Jolla Institute for Immunology, 9420 Athena Circle La Jolla, CA 92037, USA; Department of Medicine, Division of Infectious Diseases and Global Public Health, University of California, 9500 Gilman Drive MC 0507 La Jolla, CA 92093-0507, USA

## Abstract

The Immune Epitope Database (IEDB) freely provides experimental data regarding immune epitopes to the scientific public. The main users of the IEDB are immunologists who can easily use our web interface to search for peptidic epitopes via their simple single-letter codes. For example, ‘A’ stands for ‘alanine’. Similarly, users can easily navigate the IEDB’s simplified NCBI taxonomy hierarchy to locate proteins from specific organisms. However, some epitopes are non-peptidic, such as carbohydrates, lipids, chemicals and drugs, and it is more challenging to consistently name them and search upon, making access to their data more problematic for immunologists. Therefore, we set out to improve access to non-peptidic epitope data in the IEDB through the simplification of the non-peptidic hierarchy used in our search interfaces. Here, we present these efforts and their outcomes.

**Database URL:**  http://www.iedb.org/

## Introduction

The Immune Epitope Database (IEDB) (1) is a freely available database that describes structures and immunological activities associated with allergy, infectious diseases, transplantation and autoimmunity. Most of the defined epitopes, especially T-cell epitopes, are peptidic in nature. However, a significant fraction of the data (∼0.4% as of September 2020, corresponding to more than 3000 different structures) is accounted for by non-peptidic structures. Thus, while currently a minority, non-peptidic ligands provide a meaningful and growing contribution to the IEDB. To provide additional context regarding the contribution of non-peptidic ligands to the data hosted in the IEDB, we note the breakdown of non-peptidic ligands; ∼900 are associated with T-cell assays, >2500 with B-cell assays and ∼400 with MHC assays. Data for these epitopes refer to recognition in humans for almost 2000 of ligands, and data in murine systems are available for ∼1500 of ligands. Examples of these types of epitopes include lipopolysaccharide moieties recognized by serotype-specific antimicrobial antibodies, small molecules and drugs involved in allergic reactions (such as penicillin and derivatives thereof) and model haptens used in basic investigations of immune reactivity [dinitrophenyl (DNP) or alpha-galactosylceramide].

The Chemical Entities of Biological Interest (ChEBI) (2, 3), an ontological classification of ‘small’ chemical compounds, is part of the Open Biomedical Ontologies Foundry (4). The molecular entities that are considered within ChEBI’s scope can be of natural or synthetic origin, but nucleic acids and peptides/proteins are usually not in scope. The nomenclature in ChEBI relies on the International Union of Pure and Applied Chemistry (5) and the Nomenclature Committee of the International Union of Biochemistry and Molecular Biology (6). As such, ChEBI provides a relevant framework for the integration of non-peptidic molecules in the IEDB (7). The IEDB website provides a tree view of all the non-peptidic structures used in the IEDB, supported by the ChEBI structure hierarchy. These non-peptidic structures are found in the ‘chemical entity’ branch of the IEDB’s molecule tree.

The ChEBI hierarchy is organized by the chemical features of the structures. However, ChEBI contains many more terms than are required by the IEDB, and the application of ChEBI for IEDB purposes required customization to increase usability for curation, query and reporting. First, the ChEBI hierarchy did incorporate some immunological information, but this was inconsistent and not detailed enough to express the immunological information captured in the IEDB. Second, many IEDB non-peptidic structures lacked ChEBI parent structure assignments that immunologists commonly use. Third, the ChEBI tree, while rigorous, is designed primarily for chemists, and as such it was problematic for IEDB curators to navigate or for immunologists using the IEDB to find structures within the tree.

Here, we report the generation of an immunologist-friendly view of ChEBI that, while based on the ChEBI tree, was significantly streamlined and simplified specifically for IEDB applications, addressing the needs of immunologists. We generated two complementary trees: one capturing and classifying compounds by chemical structures and, new to the IEDB, a tree capturing the biological, chemical or application roles (if any) of the immunologically relevant substances, which include roles like ‘pesticide’ or ‘hormone’. All compounds contained in the role tree are also contained in the tree classifying compounds by their chemical structures; the role tree provides an alternative pathway to finding chemicals that may be more commonly known by their functions (e.g. finding ‘progesterone’ in the role tree where it is a child of ‘hormone compound’ may be easier than finding it in the structure tree where it is a child of ‘20-oxo-steroid’). Both trees are immediately visible to the user when they select the non-peptidic molecule finder in the ‘Epitope’ filter panel, after they have first selected ‘Search’ on the IEDB home page. The ‘Finder’ button is located immediately adjacent the ‘Non-peptidic’ radio button in the ‘Epitope’ filter panel on the results page. Once selected, the non-peptidic molecule finder opens as a pop-up within the IEDB, allowing users to both click through the tree manually and search via ‘Name’ or ‘Molecule ID’.

## Methods

### Glossary of ontology terminology

Throughout the paper, ontological terms are used to describe the tree classification and its attributes. Each term is defined in the glossary below to facilitate comprehension from the reader not familiar with ontological definitions, while at the same time maintaining rigor in the classification terminology. This terminology is commonly used in ontological applications (8).

**Class:** Each class within an ontology represents a type of thing in the real world, and particular instances belong to these classes. For example, your pet cat, ‘Fremont’, is an instance of the class ‘cat’, which represents the properties of all cats. ‘Cat’ is a subclass of ‘mammal’.

**Tree:** The overall subclass hierarchy of an ontology. It begins with a root class, usually ‘thing’, which represents all ‘things’ in existence. As you move down the levels of the tree, the classes become more specific.

**Grouping class:** A class that is not utilized by curated data in the IEDB but is used to help sort the tree.

**Children:** A class may have children classes using the ‘subclass of’ property. The children adopt all properties of the parent class. For example, an ‘owl’ is a child of ‘bird’ and, therefore, meets all the requirements of being a bird.

**Descendants:** The ‘subclass of’ property is transitive. A class’s child class may also have children, which still maintain all the properties of their upper-level classes. For example, ‘owl’ is a child of ‘bird’ and ‘bird’ is a child of ‘vertebrate’. Therefore, an ‘owl’ adopts all the properties of the ‘vertebrate’ class.

**Parents:** The opposite of ‘children’; a class has a parent class from which it adopts all properties.

**Ancestors:** The opposite of ‘descendants’; a class adopts all properties from all the classes above it (the ancestors).

**Siblings:** Any classes that share a parent class.

**Branch:** A specific subset of the tree based on a class and all of its descendants.

**Level:** Each ‘subclass of’ relationship creates a new level in the tree.

**Node:** Any term at any level in the hierarchy, whether it be a terminal level, without any children, or a parent level term with many children.

**Disjoint:** A class is disjoint from another class when it cannot share any instances. For example, an instance of ‘bacterium’ cannot also be an instance of ‘eukaryote’, so these two classes are disjoint.

**Roles:** ChEBI contains a role hierarchy that begins directly under the root class (‘thing’). These classes represent the types of roles that a chemical entity can have. Roles are not chemical entities, so you cannot find any chemical entities in the ‘role’ branch.

**Compounds:** We have created an alternate method of browsing ChEBI by creating a ‘compound’ hierarchy that mirrors the ‘role’ hierarchy. Chemical entities that have roles can now be found under their ‘role compound’ parent. This is explained in further detail below.

**Structural classification:** The main basis for browsing ChEBI, in which chemical entities are classified by their structures, creating a ‘structural’ hierarchy.

#### Revising and streamlining the existing ChEBI structure tree

A subset of ChEBI was created by extracting only the chemical entities curated by the IEDB and their ancestors, grouping classes such as ‘lipids’. This does not, however, limit any new chemical entities being added to the IEDB in the future. Specifically, if a chemical entity existing in ChEBI but not in the IEDB is reported to be immunogenic, the IEDB tree will be updated accordingly. This was preferred to the alternative of including all ChEBI entities prior to pruning, to reduce clutter from irrelevant, non-populated entries. A formal update process, similar to what occurs for peptidic curation, is in place.

Next, the tree was automatically pruned to remove unnecessary intermediate classes, identified as grouping classes not curated in the IEDB, and with only a limited number of other classes as children. For example, to get to ‘alpha-d glucose’ in the original ChEBI tree, it required 18 levels of grouping classes, some labeled in a manner non-intuitive for immunologists (Supplemental Figure S1a). This was reduced to the six levels (Supplemental Figure S1b), based on intuitive broad classes such as organic molecule, carbohydrate and monosaccharide. Furthermore, sparsely populated branches were condensed, and irrelevant and/or confusing grouping classes were removed. For example, ‘carbohydrates and derivatives’ were merged to create an easily browsable structure based on the number of monosaccharide units (Supplemental Figure S1c).

Finally, we created ‘other’ classes to simplify manual browsing of the tree. Any bottom-level children without a specific grouping class were placed in these ‘other’ categories. For example, ‘monosaccharide’ has 7 major grouping categories, and monosaccharides that do not fall under these categories have been placed under ‘other monosaccharide’, to ensure that the 14 extra entities do not clutter the class.

#### Usability testing

Six immunologists within the IEDB team, who had not been involved in the development of the new non-peptidic tree, were identified and requested to complete online testing via a Zoom session. The candidates were provided with de-identified web links to both the old and new non-peptidic tree and were requested to manually locate 10 different chemical entities in each tree by clicking through the branches. The automated search function was disabled and users were unable to search the internet for the chemical entities prior to locating them in the trees. Users were recorded via Zoom and timed as they searched for each entity, sharing their screen with the tester to ensure the correct entity was located. The time taken to locate each entity was recorded, and the task was ended if the entity could not be located within 3 minutes.

## Results

Currently, the IEDB contains ∼3000 non-peptidic structures. Even allowing for a 3- to 4-fold projected growth (10 000) records, it would be theoretically possible to classify these 10 000 records in 4 levels, with each class at each level containing an average of 10 children. This ideal distribution may be difficult to practically achieve, given the distribution of the records and their classification, but, nevertheless, provided a guiding principle for our efforts.

**Figure 1.**
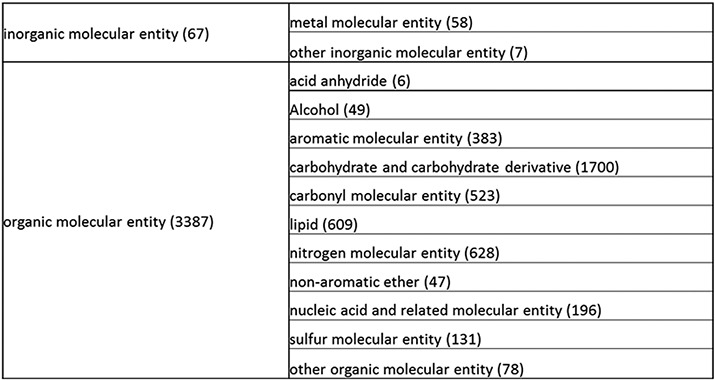
Overall structure of the revised chemical tree whereby the highest level is subdivided into organic and inorganic chemicals. The inorganic and organic branches are further subdivided into the most prominent and intuitive categories. The number of entries per level is indicated at the end of each branch’s label.

The overall simplification achieved by these processes, as described in more detail below, was remarkable. The structure tree resulting from these various modifications is shown in Figure 1. The new tree encompasses 11 level 2 branches, which are presented in Figure 2a to d. Guanosine diphosphate, abbreviated GDP (CHEBI:17552), a nucleoside phosphate, originally had 88 ancestors. In the new version of the tree, GDP has only 16 total ancestors, including structural classifications, roles and compound classifications derived from the roles. Without the roles, GDP now only has three structural ancestors, making it easier to find.

**Figure 2.**
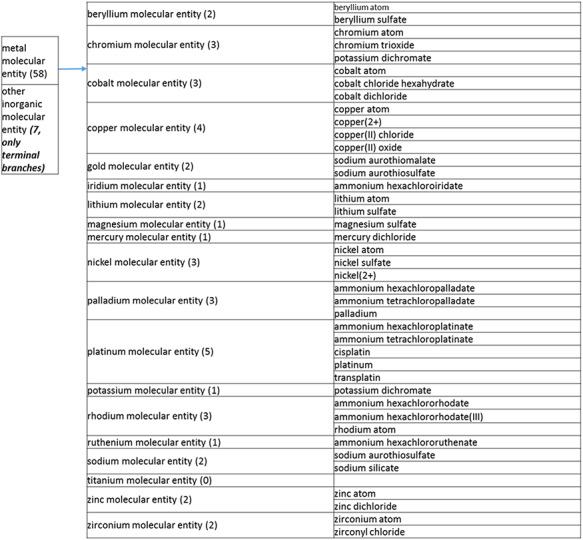
(a) A high-level breakdown of the inorganic molecular entity branch of the structure tree. Some of these are terminal branches, while others are subdivided into more specific structures for greater specificity. The number of entries per level is indicated at the end of each branch’s label.

**Figure 2.**
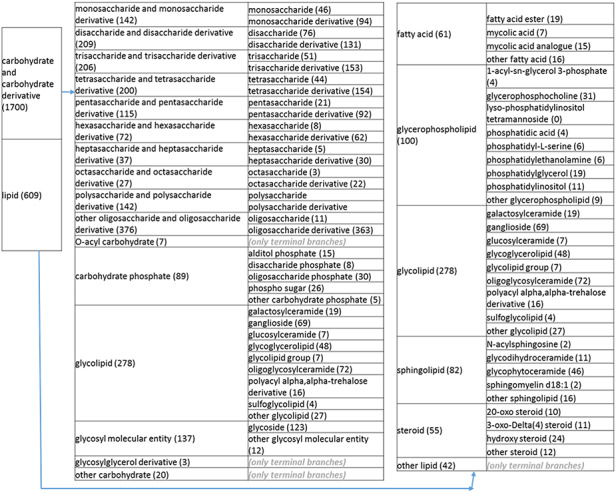
(b) A high-level breakdown of the carbohydrate and carbohydrate derivative, and lipid branches of structure tree. Both of these are children of ‘organic molecular entity’. Some of these are terminal branches, while others are subdivided into more specific structures for greater specificity. The number of entries per level is indicated at the end of each branch’s label.

**Figure 2.**
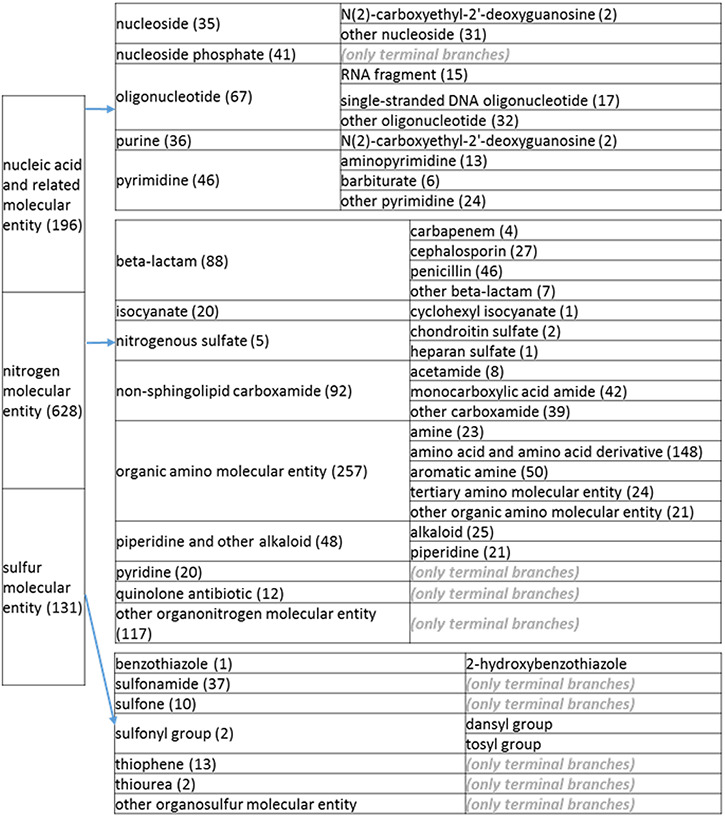
(c) A high-level breakdown of the nucleic acid and related molecular entity, nitrogen molecular entity, sulfur molecular entity, nitrogen molecular entity, sulfur molecular entity branches of the structure tree. All of these are the children of ‘organic molecular entity’ Some of these are terminal branches, while others are subdivided into more specific structures for greater specificity. The number of entries per level is indicated at the end of each branch’s label.

**Figure 2.**
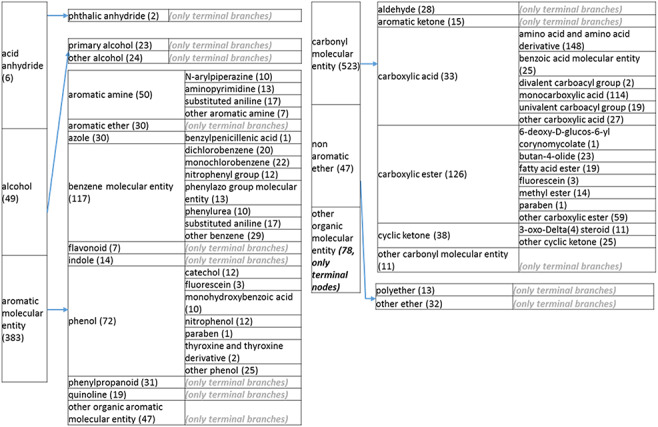
(d) A high-level breakdown of the acid an hydride, alcohol, aromatic molecular entity, carbonyl molecular entity, non-aromatic ether and other molecular entity branches of the structure tree. All of these are children of ‘organic molecular entity’. Some of these are terminal branches, while others are subdivided into more specific structures for greater specific structures for greater specificity. The number of entries per level is indicated at the end of each branch branch’s label.

During this process, limiting redundancies was another goal. ChEBI classifies structures based on their chemical determinants. For example, it is intuitive to allow access to a glycolipid from both the lipid and carbohydrate branches. However, strictly speaking, any organic compound in which the hydroxyl functional group (–OH) is bound to a carbon is considered an alcohol, and to classify carbohydrates as alcohols because of the presence of an –OH group is counterintuitive to immunologists. It also leads to a cumbersome tree for browsing and maintaining. Accordingly, we arbitrarily eliminated some of these occurrences based on our judgment of what users would expect.

### Streamlining the structure tree, by removing ‘mixtures’, ‘groups’ and ‘derivatives’

A high-level branch in the ChEBI tree is dedicated to a ‘mixture’, which is defined as ‘a chemical substance composed of multiple molecules, at least two of which are of a different kind’. This was not a very useful category for our purposes. We accordingly streamlined the structure tree; Supplemental Table S1 provides a summary of the group/derivative types and the changes. Most entries were duplicated elsewhere, and the few (about 15) ChEBI terms used in the IEDB found only under ‘mixture’ were relocated. For example, in the case of a glycolipid (which was captured as a mixture of a glyco and a lipid), we duplicated entries and created a node for sugars that contain lipids under the sugar branch, and reciprocally created a ‘sugar that contains lipids’ node in the lipid branch. An additional example is provided by neomycin sulfate. In this case, the sulfate is a salt and is not part of the immunologically recognized structure. The amino sugar node was used to capture the neomycin molecule.

Likewise, the categories ‘groups’ or ‘derivatives’ encompassed records relating to a prominent part of the molecule that would be most logically found as part of the node describing the molecule. All groups were also relocated. Amino sugars were placed under amino [prefix]saccharide group, under their respective amino [prefix]saccharides. We created a nitrophenyl (NIP) group branch under benzene molecular entity to include a trinitrophenyl group, DNP group, o-nitrophenyl group and p-nitrophenyl group. Phenylazo groups and its children were also moved under benzene molecular entity. The penicilloyl group and its children were placed under penicillin, which now also includes the amoxicilloyl, ampicilloyl and benzylpenicilloyl groups. Amino acid derivatives were placed under their respective amino acids, e.g. *N*-acetyl-alpha-D-galactosaminyl-l-serine residue is placed under ‘serine and serine derivative’. Sugar groups under ‘other glycosyl group’ were placed under ‘other glycosyl [prefix]saccharide group’, unless they contain aminyl groups, in which case they were placed under their respective amino sugar groups. Tosyl and dansyl groups were moved to ‘other benzene’, and, additionally, we created a new node for them called ‘sulfonyl group’ under ‘sulfur molecular entity’. The univalent carboacyl group and its 19 children were placed under ‘carboxylic acid’, since ChEBI defines univalent carboacyl group as ‘a group formed by loss of OH from the carboxy group of a carboxylic acid’. However, some of its members were also placed under other groups; for example, ‘amoxicilloyl group’ is under ‘penicilloyl group’.

Three NIP-related compounds that were under the ‘univalent carboacyl group’ were also placed into a new node below the ‘nitrophenyl group’, called ‘nitrophenylacetyl group’. A few new nodes were created to accommodate special situations. For example, a node for the ‘phenylazo group’ was created under ‘nitrogen molecular entity’, and a node for the ‘carboacyl group’ was created under ‘organic molecular entity’.

### Streamlining the structure tree, by reorganizing the structures into more intuitive formats

Next, we returned to the high-level organics nodes to eliminate errors and reorganize the structures to more intuitive formats. In reviewing the ‘carbohydrates and derivatives’ branches, we noted the presence of glycopeptides that are mostly peptides with modifications. This reflected a curation mistake and prompted us to remove the glycopeptides from the non-peptidic tree and recurate the papers. Posttranslational modifications of peptides are indeed curated in the IEDB and accessible through the associated peptidic moiety, with the exception of records relating to recognition of amino acids in isolation. We also noted several glycolipids and phospho sugars. These were placed in separate branches on the same level as monosaccharides and their derivatives, disaccharides and their derivatives, etc. Glycolipid groups with varying numbers of sugar moieties were placed in a new group called ‘glycolipid group’ under ‘glycolipid’.

Another example of reorganization/simplification related to ‘ethers’: ‘aromatic ethers’ were found both as a branch in ‘aromatic’ and ‘ethers’. We deleted aromatic ethers from ethers and renamed the ‘ethers’ node to ‘non-aromatic ether’. We moved all phosphatidylcholine entities under a single branch below ‘glycerophosphocholine’, removing the level 4 phosphatidylcholine branch under lipids and the level 2 phosphatidylcholine(1+) branch under nitrogen compounds.

Significant effort was also devoted to restructuring amino acid nodes. We implemented the following organizational structure, using alanine as an example amino acid: organic amino compound (level 2); amino acid (level 3); alanine and alanine derivatives (level 4). All 20 natural amino acids were kept at the same level. Under the nitrogen compounds, glycopeptides and oligopeptides were both under ‘amino acid derivative’ and ‘carboxamide’. The ‘carboxamide’ affiliation was eliminated. Leukotriene E4 was preserved under amino acids because it contains a cysteine, but was also retained under ‘other fatty acids’, which is a more intuitive placement. Citrulline was placed in ‘other amino acids’.

Additional redundancies within the nitrogen branch included galactosylceramide, ganglioside, oligoglycosylceramide, glucosylceramide, glycodihydroceramide, glycophytoceramide, *N*-acylsphingosine and sphingomyelin, which were under both the carboxamide branch and the sphingolipid branch. We eliminated the redundancies; retained *N*-acylsphingosine, glycohydroceramide, glycophytoceramide and sphingomyelin under ‘sphingolipid’; retained galactosylceramide, ganglioside, glucosylceramide and oligoglycosylceramide under ‘glycolipid’ and renamed the carboxamide branch ‘non sphingolipid carboxamide’. For the acetamide branch (level 3), ‘other acetamide’ was removed as a sub-branch because acetamide only had eight members. Likewise, ‘other isocyanate’ was removed as a sub-branch for similar reasons. Penicilloic acid was removed as a level 2 branch because it was already found under penicillin. Chondroitin sulfate and heparan sulfate were consolidated into one level 2 branch called ‘nitrogenous sulfate’ because of being sparsely populated.

### The final product

In the early stages of the reorganization, ‘other organic molecular entity’ was created as a placeholder for entities whose placement was not immediately apparent or intuitive. After these revisions, we returned to the ‘other’ categories, trying to place as many as possible under appropriate branches. In this work, we also relied heavily on logical definitions provided in the ChEBI tree. In the end, the ‘other molecular entity’ category only contained 78 structures as compared to 135 when we started working on the placements.

We also reviewed the tree for consistency between plural and singular names (pyridines vs pyridine). We followed the recommended convention for ontologies and controlled vocabularies to use lower case, unless the word would be capitalized in the middle of a regular sentence (names, atom symbols, abbreviations, etc.), and to use the singular form. Other names in the tree were inconsistent, between using ‘compounds’, ‘atom or molecule’ and ‘molecular entity’. We settled on using the term ‘molecular entity’, defined as ‘any constitutionally or isotopically distinct atom, molecule, ion, ion pair, radical, radical ion, complex, conformer, etc., identifiable as a separately distinguishable entity’. This also allows the use of the two broadest terms as ‘inorganic molecular entity’ and ‘organic molecular entity’.

The tree was once again inspected for unnecessary redundancies, which were eliminated. As a design goal, no level 2 nodes should be repeated, although we did make an exception for glycolipids, which are in both lipids and carbohydrates. ‘Other’ nodes are always listed last among their siblings. Prior to reorganization, the IEDB’s non-peptidic epitope finder was difficult to navigate, and our goal for a finished product was a tree that was both markedly more streamlined and more intuitive for an immunologist to use. Through the iterative simplification process described above, we have created a product that is demonstrably easier to navigate for an end user.

The structure tree resulting from the various modifications described above is shown in Figure 1. The high level starts by subdividing the tree in organic and inorganic molecules. In the inorganic branch, metals are a prominent category and also incorporate instances where the metal was the most prominent feature (like derivatives and groups containing a metal atom). In the organic branch, the reorganized, more intuitive level 1 structure includes (i) acid anhydride, (ii) alcohol, (iii) aromatic molecular entity, (iv) carbohydrate and carbohydrate derivative, (v) carbonyl molecular entity, (vi) lipid (including phosphatidylcholine as a sub-branch), (vii) nitrogen molecular entity, (viii) non-aromatic ether, (ix) nucleic acid and related molecular entity (to include all level 2 nodes in nucleoside, nucleoside phosphate, oligonucleotide and purine), (x) sulfur molecular entity and (xi) other organic molecular entities. A high-level breakdown of the 11 level 2 branches is also presented in Figure 2a to d.

### Quantifying simplification

The non-peptidic tree shown above is measurably simpler than the old tree along two key axes. First, the old tree is much deeper than the new tree, meaning that the new tree has fewer layers that users must navigate as they search for specific non-peptides. Table 1 shows that the maximum depth of the old tree is 23 levels, while the maximum depth of the new tree is just 9. Likewise the average and median depths for the new tree are half of those measures for the old tree.

**Table 1.** Quantitative data showing the simplified nature of the new non-peptidic tree, as compared to the old tree

	Old tree	New tree
Average source depth	12.3	5.6
Median source depth	12	6
Maximum source depth	23	9
Minimum source depth	3	3
Average source count per parent	3.5	10.2
Median source count per parent	1	3
Maximum source count per parent	274	234
Parents with one source child	838	135

The trees have been compared based on their depth and source count per cent.

Second, the new tree is also ‘broader’ on average, with more source nodes per parent node and fewer parents with just one child. In the old tree, users would often have to ‘dig down’ many levels, where each level consisted of just one new child node. In fact, the median number of source nodes per parent node in the old tree is just 1 and the average is just 3.5. In the new tree, the median number of source nodes per parent node is 3 and the average is also much higher at 10.2.

We argue that it is important to strike a balance between the depth and the breadth of a tree. A ‘tree’ that consists of 100 nodes at the first level has a minimal depth of 1, but the user does not benefit from useful grouping nodes. On the other hand, grouping nodes are of little use when the ‘group’ is just a single node. The new tree strikes a better balance than the old tree, by making the average and maximum depths more manageable for users, while also ensuring that grouping nodes are more effective.

### Revising and streamlining the existing ChEBI role tree

The next set of activities was aimed at developing the role tree. As mentioned above, the IEDB curates the immunological data associated with each epitope structure, whether peptidic or not. ChEBI contains some information about certain structures being allergens, haptens or otherwise immunogenic by assigning roles to the chemical entities. Much of this work comes from past collaborations with the IEDB, but this was done at static time points and was not well maintained. Thus, this information was not rigorously controlled, neither at the level of vocabulary nor with actual evidence supporting the attribution. This created potential for data inconsistencies and user confusion.

Thus, the ‘role’ branch was reviewed and revised to group roles commonly used by immunologists to better suit the needs of the IEDB. For example, many lower-level role categories were simplified. Roles in ChEBI are typically asserted in the pattern ‘structure subClassOf “has role” some role’ (e.g. ‘amoxicillin subClassOf “has role” some “antibacterial drug”’). While providing useful information, these anonymous parents, named because they do not reference a distinct entity within the ontology, are not browsable. To make it possible to browse for a chemical entity by its role, we created a ‘chemical entity by role’ class as a sibling to the ‘chemical entity’ class. The role hierarchy duplicates the structure hierarchy as ‘X compound’ (e.g. ‘antimicrobial drug’), and chemical entities can now be found under their respective role parent. Supplemental Figure S2a shows how ‘amoxicillin’ can be found under ‘antibacterial drug’. Structurally, ‘amoxicillin’ can also be found under ‘penicillin’ as shown in Supplemental Figure S2b.

### Identification of curated compounds with no assigned ChEBI role

While ChEBI does include many ‘role’ axioms, not all chemical entities have a defined role. This may be because ChEBI has not yet curated them, or it is not within the scope of ChEBI. As discussed above, ChEBI asserts roles using the pattern ‘structure subClassOf “has role” some role’. In order to be a part of the ‘compound’ hierarchy, a structure or one of its ancestor structures must have this axiom. Ideally, each chemical entity curated within the IEDB should be inspected for possible biological roles.

We identified those structures missing a defined role by querying the revised ChEBI tree using SPARQL, a query language that can be used on ontologies. First, we only looked at structures that did not have a good structural parent (those that were IEDB-curated and appeared in a ‘other’ grouping class) and found 464 entities. Once this review was complete, we expanded the query to include any chemical entity without a role. This returned 1873 additional entities to which we assigned roles using the process below.

### Role review and assignment and generating new roles and placement in the role tree

All chemicals in ChEBI that did not have a defined non-immunological role were exported into an Excel spreadsheet. Each row consisted of the ChEBI ID, a link to the corresponding epitope on the IEDB, the name of the compound and the ontological ‘parents’ or grouping classes. We then identified one or more roles for each compound using information in the manuscript(s) that chemical appeared in databases like PubChem and/or internet searches. Once these roles had been identified, they were entered in the spreadsheet as short text descriptions.

Of the 2333 chemicals from the Excel spreadsheet mentioned above, we found that a total of 1623 compounds were synthesized/studied and curated in the IEDB only because they stimulate, or are recognized by, an immune response. These included, but are not limited to, mammalian or microbial carbohydrates, oligonucleotides and haptens. For these compounds, we captured a NR (No Role) in the short text description to indicate that they were reviewed and that there was no role to be attached to them and eliminated them from the simplified role tree. Clear roles were identified for the remaining 710 chemicals. These roles included antibiotics and other medications, industrial materials, dyes, food additives, pesticides and reagents, among others.

Following completion of the review of all compounds with no previous role assignments, we reviewed the short text role descriptions. If suitable roles existed in the revised ChEBI tree mentioned above, the compounds were assigned existing roles. If a suitable role was not identifiable, new roles were added under in the ‘role’ branch in their appropriate category (application, biological or chemical).

### The revised role tree

The role tree resulting from the various modifications described above is shown in Figure 3a to d. At the highest level, the tree is subdivided into three separate role classes, namely the ‘application’ (Figure 3a and b), the ‘biological role’ (Figure 3c) and the ‘chemical role’ (Figure 3d) branches. Each branch is further subdivided into level 2 branches. Level 2 branches under ‘application’ are cosmetic or detergent; drug; dye, probe and color-related application; excipient; food and environmental application; manufacturing and industrial application; photography and imaging agent; preservative and reagent (Figure 3a and b). Level 2 branches under ‘biological role’ are acetylcholine-releasing agent, advanced glycation end product, agonist, antagonist, antimutagen, carcinogenic and toxic agent, disinfectant, endocrine disruptor, growth regulator, immunomodulator, inhibitor, lipid peroxidation end product, mitogen, transition state analog and other biochemical role (Figure 3c). Finally, level 2 branches under ‘chemical role’ are acid, antioxidant, base, buffer, chain carrier, corrosion inhibitor, environmental contaminant, solvent, other chemical role and other experimental carbohydrate and non-peptidic (Figure 3d).

**Figure 3.**
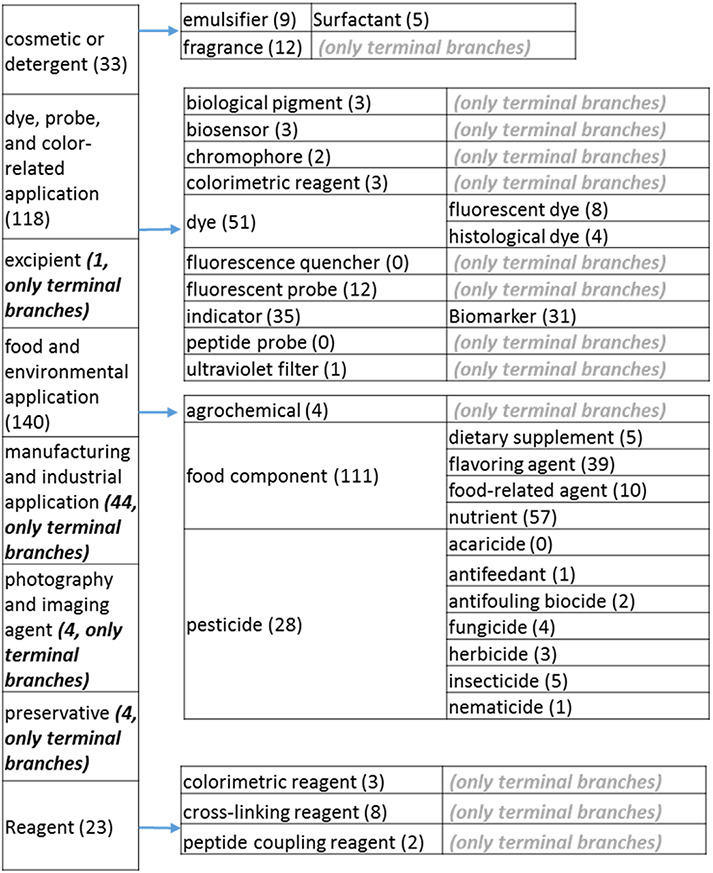
(a) The ‘application’ role class of the revised role tree, excluding the drug branch, which is further subcategorized into the most prominent application. Some of these are terminal branches, while others are subdivided into more specific application types for greater specificity. The number of entries per level is indicated at the end of each branch’s label.

**Figure 3.**
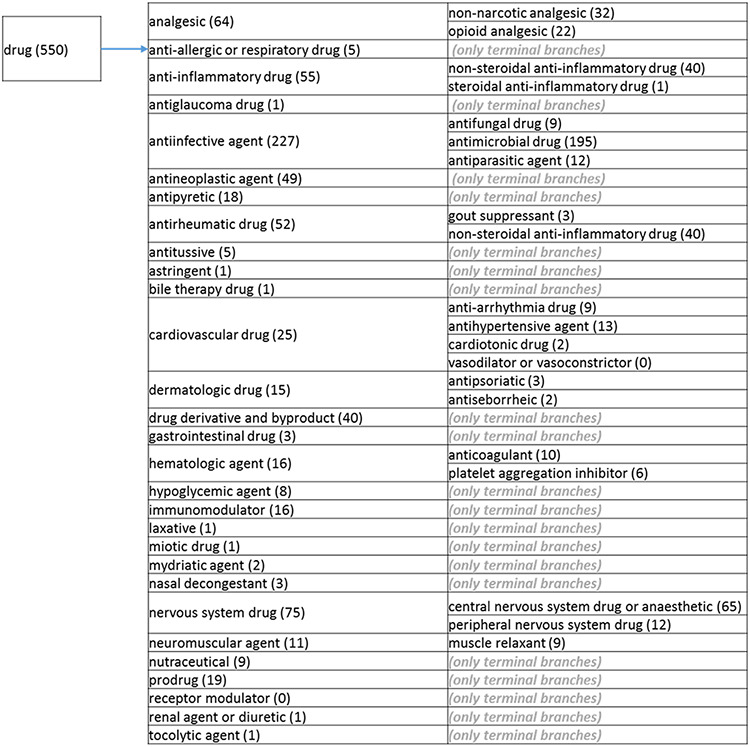
(b) The ‘drug’ branch of the ‘application’ role class of the revised role tree, which is further subcategorized into the most prominent applications. Some of these are terminal branches, while others are subdivided into more specific application types for greater specificity. The number of the entries per level is indicated at the end of each branch’s label.

**Figure 3.**
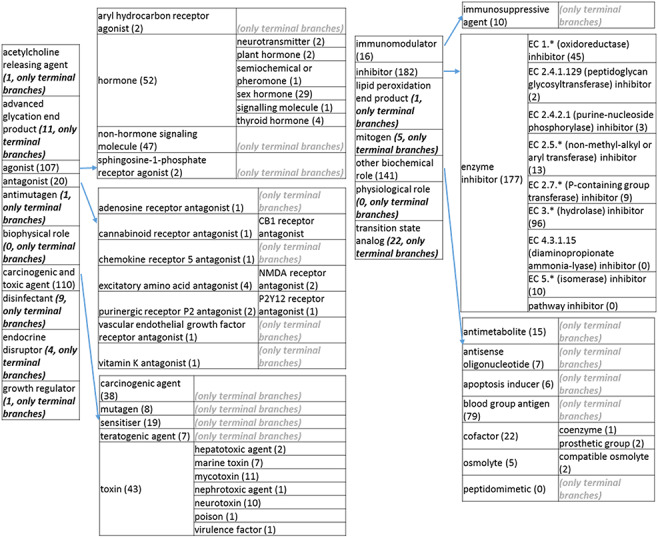
(c) The ‘biological role classes of the revised role tree, which is further subcategorized into the most prominent biological functions. Some of these are terminal branches, while others are subdivided into more specific biological role types for greater specificity. The number of entries per level is indicated at the end of each branch’s label.

**Figure 3.**
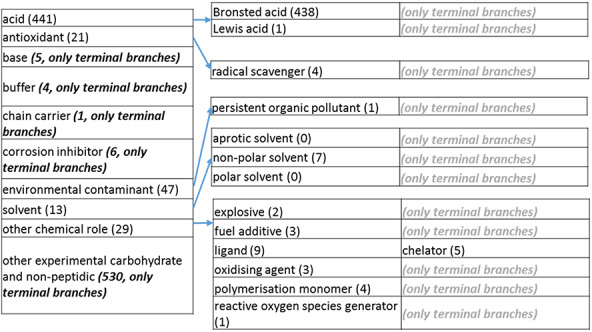
(d) The ‘chemical role’ role class of the revised role tree, which is further subcategorized into the most prominent chemical roles. Some of these are terminal branches, while others are subdivided into more specific chemical roles. Some of these are terminal branches, while others are subdivided into more specific chemical role types for greater specificity. The number of number of entries per level is indicated at the end of each branch’s label.

Likewise, ChEBI inconsistently curates the organisms from which the various chemical entities are derived. This is due to only capturing such information when it was present in the original source used for the ChEBI entry. For example, if a ChEBI structure was created from a publication describing it as being derived from a specific species of bacteria, that bacterial source will be included in the entry. However, if that same chemical structure is present in several other species of bacteria that were not mentioned by the publication, ChEBI may not include references to the additional bacterial species in their curated record. The IEDB captures taxonomic information in specific fields that are maintained and linked to the NCBI Taxonomy (9). Accordingly, the information from the IEDB was used to assign source organisms for ChEBI chemical entities and create a browsable source organism hierarchy. This will allow for an alternative method of browsing. All non-peptidic records were reviewed, and many of the non-peptidic curations were reviewed and edited to ensure that taxonomic information associated with IEDB curated data was entered and accurate. At the same time, roles based on taxonomy were eliminated from the tree.

### Process for maintaining and updating the tree and roles

If our process identified errors or missing structural parents or roles for non-peptidic structures, we sent this information to a ChEBI curator, so that these changes could be made to the actual ChEBI entries. This was actually rare, with most of the effort of this project being a simplification and reduction of the utilizing content present in the ChEBI entries. Once changes were available in the ChEBI website, a new version of the ChEBI ontology was retrieved and our tree-pruning routines, as described above, were run upon it. The result is a new immunologically friendly ChEBI hierarchy with a heavily streamlined structure branch geared toward immunologists.

As we encounter new non-peptidic structures that are not already present in our streamlined tree, we first search the ChEBI website to determine if the structure already exists in ChEBI. If it does, we add it, along with information on which of the many structural parents is most immunologically relevant to the IEDB via direct addition to our simplified ontology file that the IEDB’s molecule tree utilizes. At this time, we also review any roles assigned to the structure and provide input to the ChEBI team, if necessary. If the structure does not yet exist in ChEBI, we request its addition via a new term request made to a ChEBI curator.

In order to remain up to date with changes in the ChEBI content, the weekly build process of the IEDB incorporates a validation step that checks if all ChEBI structures in our data are still valid. If any structure is not valid, we check for a replacement term in ChEBI. If such a term is not found, we manually inspect and correct the structure.

### Usability testing

**Figure 4.**
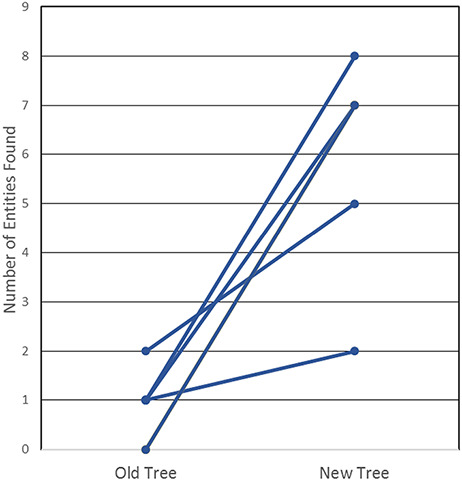
User testing comparison of old and new non-peptidic trees, where users were tasked in locating 20 non-peptidic entities across the two trees while being timed. Statistical significance was achieved with *P* < 0.005, as all participants consistently located more entities in the new tree.

In order to determine whether this new tree was, indeed, more useful and intuitive, we then tested our curators and end users, all immunologists who had not been involved in the development of the revised tree. We created a test that required the prospective user to find 20 common non-peptidic structures by manually navigating both the old and the new ChEBI trees; they were asked to find half in the old tree and the other half in the new tree. The test that was conducted is provided in Supplemental Table S2. The results of the test, as shown in Figure 4, highlight that the new, revised hierarchies are clearly superior for use by immunologists, whereby all six users were consistently able to find more epitopes in the new tree when compared with the old tree. In fact, participants were only able to locate a maximum of two structures in the old tree within the provided 3-minute window, with three of the participants locating only one structure, while two participants were unable to locate any of the structures. In contrast, participants were able to locate up to 8 of the 10 non-peptidic structures in the new tree, well within the 3-minute window and with no assistance from Google, a staggering increase from a usability perspective.

## Discussion

By using the ChEBI resource to depict non-peptidic structures in the IEDB, we were able to start with a great deal of specialized chemical knowledge, which we were able to prune down to meet the needs of our specific immunologist community. The searchability of non-peptidic data was improved in the IEDB by removing extraneous nodes, simplifying the hierarchy of epitope structures, and by making immunologist-relevant roles available as a new search feature. This work clearly decreased the complexity of the non-peptidic search, as seen by the total numbers of nodes and depth present in the new tree. Through user testing, we were also able to demonstrate significant improvements in the access to non-peptidic epitope data in the IEDB. This initial testing was based on internal users, and our future plans include gathering external user feedback to determine further effectiveness or refinement, following the same process that has been utilized to generally refine IEDB query and reporting processes (9). Based on the simplification and reorganization of the non-peptidic classification, we plan to increase our curation of non-peptidic ligands, and, accordingly, we plan to publish a separate study addressing in detail the nature and roles of immune recognition of non-peptidic ligands. We hope that this work will benefit the users of the IEDB, and, as a free public resource, we will continue to seek feedback from our user community.

## Supplementary Material

baab014_Supp

## References

[1] Vita,R., Mahajan,S., Overton,J.A. et al. (2019) The Immune Epitope Database (IEDB): 2018 update. *Nucleic Acids Res.*, 47, D339–D343. 30357391 10.1093/nar/gky1006 PMC6324067

[2] De Matos,P., Alcantara,R., Dekker,A. et al. (2010) Chemical entities of biological interest: an update. *Nucleic Acids Res.*, 38, D249–D254. 19854951 10.1093/nar/gkp886 PMC2808869

[3] Degtyarenko,K., De Matos,P., Ennis,M. et al. (2008) ChEBI: a database and ontology for chemical entities of biological interest. *Nucleic Acids Res.*, 36, D344–D350. 17932057 10.1093/nar/gkm791 PMC2238832

[4] Smith,B., Ashburner,M., Rosse,C. et al. (2007) The OBO Foundry: coordinated evolution of ontologies to support biomedical data integration. *Nat. Biotechnol.*, 25, 1251–1255. 17989687 10.1038/nbt1346 PMC2814061

[5] Favre,H.A. and Powell,W.H. (2014) *International Union of Pure and Applied Chemistry Nomenclature of Organic Chemistry: IUPAC Recommendations and Preferred Names 2013*. Royal Society of Chemistry, Cambridge.

[6] Liébecq,C. (1992) *Biochemical Nomenclature and Related Documents: A Compendium*. Portland Press, London.

[7] Vita,R., Peters,B., Josephs,Z. et al. (2011) A model for collaborative curation, the IEDB and ChEBI curation of non-peptidic epitopes. *Immunome. Res.*, 7, 1–8.

[8] Hastings,J., Magka,D., Batchelor,C. et al. (2012) Structure-based classification and ontology in chemistry. *J. Cheminform.*, 4, 8. 10.1186/1758-2946-4-8 PMC3361486 22480202

[9] Federhen,S. (2012) The NCBI taxonomy database. *Nucleic Acids Res.*, 40, D136–D143. 22139910 10.1093/nar/gkr1178 PMC3245000

