# Supplementary material for: An immunologically friendly classification of non-peptidic ligands
Source: Database (Oxford). 2021 Mar 27;2021:baab014. doi: 10.1093/database/baab014 (PMC8001080; doi:10.1093/database/baab014)
Supplement: baab014_Supp [file baab014_supp.zip › Non-peptidic Manuscript - Supplemental Figure 1a (Resubmission).docx]

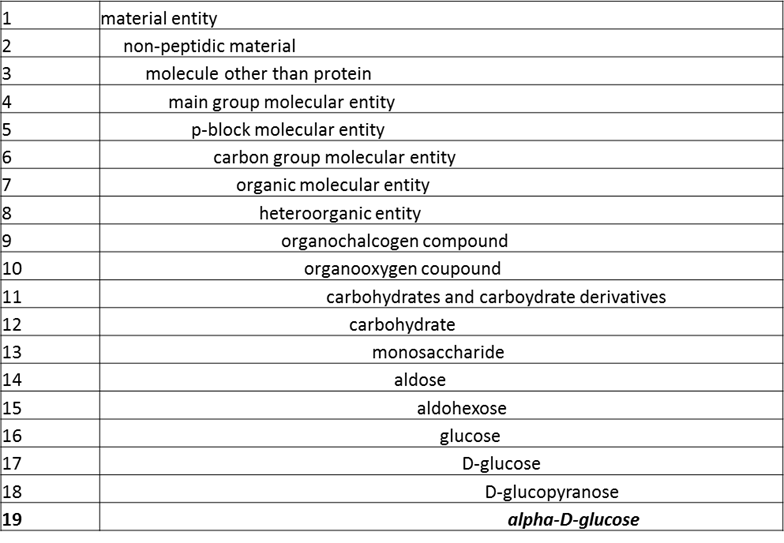


**Supplemental Figure 1a.** The location of ‘alpha-D-glucose’ in the original ChEBI tree. In this unrevised version, finding alpha-D-glucose requires the user to click through 18 levels, many of which are unintuitive to immunologists.
