# Supplementary material for: An immunologically friendly classification of non-peptidic ligands
Source: Database (Oxford). 2021 Mar 27;2021:baab014. doi: 10.1093/database/baab014 (PMC8001080; doi:10.1093/database/baab014)
Supplement: baab014_Supp [file baab014_supp.zip › Non-peptidic Manuscript - Supplemental Figure 1c (Resubmission).docx]

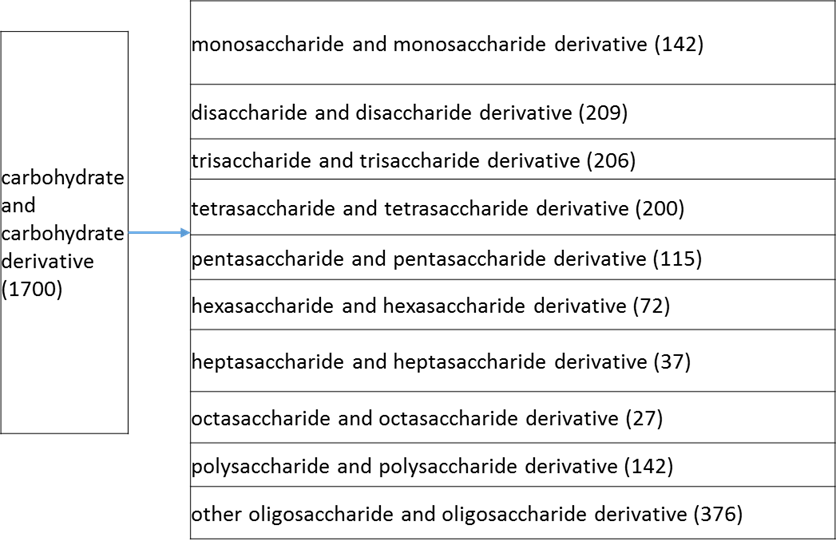


**Supplemental Figure 1c.** The high-level organizational structure of the carbohydrate branch of the revised ChEBI tree, where structures are organized by the number of carbohydrate moieties. The carbohydrate and carbohydrate derivative branch also has sub-branches that organize chemical entities by more specific structures; these branches are O-acyl carbohydrate, carbohydrate phosphate, glycolipid, glycosyl molecular entity, glycosylglycerol derivative, and other carbohydrate. They are sibling branches to those organized by carbohydrate moiety, and a more comprehensive view of the carbohydrate and carbohydrate derivative branch can be found in **Figure 2b**. The number of entries per level is indicated at the end of each branch’s label.
