# Supplementary material for: An immunologically friendly classification of non-peptidic ligands
Source: Database (Oxford). 2021 Mar 27;2021:baab014. doi: 10.1093/database/baab014 (PMC8001080; doi:10.1093/database/baab014)
Supplement: baab014_Supp [file baab014_supp.zip › Non-peptidic Manuscript - Supplemental Table 1 (Resubmission).docx]

**Supplemental Table 1.** Relocation of all entities under the category ‘groups’ in the old non-peptidic tree.

| **Group in old tree** | **Location of group in revised tree** |
| --- | --- |
| group | Removed |
| organic group | Removed |
| glycosyl group | Removed |
| D-glucosyl group | Placed under new branch ‘other glycosyl monosaccharide group’ |
| alpha-Neup5Ac-(2->8)-alpha-Neup5Ac-(2->3)-beta-D-Galp-(1->4)-beta-D-Glcp-yl group | Placed under new branch ‘amino tetrasaccharide group’ |
| alpha-D-galactosyl group | Placed under new branch ‘other glycosyl monosaccharide group’ |
| alpha-L-Fuc-(1->2)-beta-D-Gal-(1->4)-beta-D-GlcNAc-(1->3)-alpha-D-Gal-yl group | Placed under new branch ‘amino tetrasaccharide group’ |
| glucosaminyl group | Removed |
| alpha-D-Galp-(1->3)-beta-D-Galp-(1->4)-D-GlcpNAc-yl group | Placed under new branch ‘amino trisaccharide group’ |
| alpha-D-Galp-(1->3)-beta-D-Galp-(1->4)-beta-D-GlcpNAc-yl group | Placed under new branch ‘amino trisaccharide group’ |
| beta-D-GalpNAc-(1->4)-[alpha-L-Fucp-(1->3)]-D-GlcpNAc-yl group | Placed under new branch ‘amino trisaccharide group’ |
| N-acetyl-beta-D-galactosaminyl-(1->4)-[alpha-L-fucosyl-(1->3)]-N-acetyl-beta-D-glucosaminyl group | Placed under new branch ‘amino trisaccharide group’ |
| other glucosaminyl group | Removed |
| 3-O-{(1R)-1-[(1,3-dihydroxypropan-2-yl)oxy]-2-hydroxyethyl}-2,6-bis-O-{(1S)-1-[(1,3-dihydroxypropan-2-yl)oxy]-2-hydroxyethyl}-beta-D-Man-(1->4) -beta-D-GlcNAc-(1->4)-3-O-[(1S)-2-hydroxy-1-{[(2S)-1-hydroxypropan-2-yl]oxy}ethyl]-D-GlcNAc-yl group | Placed under new branch ‘amino trisaccharide group’ |
| N-acetyl-beta-D-galactosaminyl-(1->4)-N-acetyl-beta-D-glucosaminyl group | Placed under new branch ‘amino disaccharide group’ |
| alpha-D-Manp-(1->2)-alpha-D-Manp-(1->2)-alpha-D-Manp-(1->3)-beta-D-Manp-(1->4)-beta-D-GlcpNAc-(1->4)-D-GlcpNAc-yl group | Placed under new branch ‘amino hexasaccharide group’ |
| alpha-D-Manp-(1->3)-[alpha-D-Manp-(1->6)]-beta-D-Manp-(1->4)-beta-D-GlcpNAc-(1->4)-D-GlcpNAc-yl group | Placed under new branch ‘amino pentasaccharide group’ |
| alpha-L-Fuc-(1->2)-alpha-L-Fuc-(1->3)-beta-D-GalNAc-(1->4)-[alpha-L-Fuc-(1->2)-alpha-L-Fuc-(1->3)]-beta-D-GlcNAc-yl group | Placed under new branch ‘amino hexasaccharide group’ |
| alpha-L-Fuc-(1->3)-beta-D-GalNAc-(1->4)-[alpha-L-Fuc-(1->3)]-beta-D-GlcNAc-yl group | Placed under new branch ‘amino tetrasaccharide group’ |
| alpha-L-Fuc-(1->3)-beta-D-GalNAc-(1->4)-beta-D-GlcNAc-yl group | Placed under new branch ‘amino trisaccharide group’ |
| alpha-L-Fucp-(1->2)-[alpha-D-Galp-(1->3)]-beta-D-Galp-(1->3)-beta-D-GlcpNAc-yl group | Placed under new branch ‘amino tetrasaccharide group’ |
| alpha-L-Fucp-(1->2)-[alpha-D-Galp-(1->3)]-beta-D-Galp-(1->4)-beta-D-GlcpNAc-yl group | Placed under new branch ‘amino tetrasaccharide group’ |
| alpha-L-Fucp-(1->2)-[alpha-D-GalpNAc-(1->3)]-beta-D-Galp-(1->3)-beta-D-GlcpNAc-yl group | Placed under new branch ‘amino tetrasaccharide group’ |
| alpha-L-Fucp-(1->2)-[alpha-D-GalpNAc-(1->3)]-beta-D-Galp-(1->4)-beta-D-GlcpNAc-yl group | Placed under new branch ‘amino tetrasaccharide group’ |
| alpha-L-Fucp-(1->2)-beta-D-Galp-(1->3)-[alpha-L-Fucp-(1->4)]-beta-D-GlcpNAc-yl group | Placed under new branch ‘amino tetrasaccharide group’ |
| alpha-L-Fucp-(1->2)-beta-D-Galp-(1->3)-beta-D-GlcpNAc-yl group | Placed under new branch ‘amino trisaccharide group’ |
| alpha-L-Fucp-(1->2)-beta-D-Galp-(1->4)-[alpha-L-Fucp-(1->3)]-beta-D-GlcpNAc-yl group | Placed under new branch ‘amino tetrasaccharide group’ |
| alpha-L-Fucp-(1->3)-[beta-D-Galp-(1->4)]-beta-D-GlcpNAc-(1->2)-alpha-D-Manp-(1->3)-[beta-D-Galp-(1->4)-beta-D-GlcpNAc-(1->2)-alpha-D-Manp-(1->6)]-beta-D-Manp-(1->4)-beta-D-GlcpNAc-(1->4)-D-GlcpNAc-yl group | Placed under new branch ‘amino oligosaccharide group’ |
| alpha-L-Fucp-(1->3)-[beta-D-Galp-(1->4)]-beta-D-GlcpNAc-yl group | Placed under new branch ‘amino trisaccharide group’ |
| alpha-L-Fucp-(1->3)-beta-D-GalpNAc-(1->4)-[alpha-L-Fucp-(1->3)]-D-GlcpNAc-yl group | Placed under new branch ‘amino tetrasaccharide group’ |
| alpha-Neup5Ac-(2->3)-beta-D-Galp-(1->4)-beta-D-GlcpNAc-(1->2)-[alpha-Neup5Ac-(2->3)-beta-D-Galp-(1->4)-beta-D-GlcpNAc-(1->4)]-alpha-D-Manp-(1->3)-beta-D-Manp-(1->4)-beta-D-GlcpNAc-(1->4)-D-GlcpNAc-yl group | Placed under new branch ‘amino oligosaccharide group’ |
| alpha-Neup5Ac-(2->6)-beta-D-Galp-(1->4)-beta-D-GlcpNAc-(1->2)-[alpha-Neup5Ac-(2->6)-beta-D-Galp-(1->4)-beta-D-GlcpNAc-(1->4)]-alpha-D-Manp-(1->3)-[alpha-Neup5Ac-(2->6)-beta-D-Galp-(1->4)-beta-D-GlcpNAc-(1->2)-alpha-D-Manp-(1->6)]-beta-D-Manp-(1->4)-beta-D-GlcpNAc-(1->4)-D-GlcpNAc-yl group | Placed under new branch ‘amino oligosaccharide group’ |
| alpha-Neup5Ac-(2->6)-beta-D-Galp-(1->4)-beta-D-GlcpNAc-(1->2)-[alpha-Neup5Ac-(2->6)-beta-D-Galp-(1->4)-beta-D-GlcpNAc-(1->4)]-alpha-D-Manp-(1->3)-[alpha-Neup5Ac-(2->6)-beta-D-Galp-(1->4)-beta-D-GlcpNAc-(1->6)-[beta-D-Galp-(1->4)-beta-D-GlcpNAc-(1->2)]-alpha-D-Manp-(1->6)]-beta-D-Manp-(1->4)-beta-D-GlcpNAc-(1->4)-D-GlcpNAc-yl group | Placed under new branch ‘amino oligosaccharide group’ |
| alpha-Neup5Ac-(2->6)-beta-D-Galp-(1->4)-beta-D-GlcpNAc-(1->2)-[alpha-Neup5Ac-(2->6)-beta-D-Galp-(1->4)-beta-D-GlcpNAc-(1->4)]-alpha-D-Manp-(1->3)-beta-D-Manp-(1->4)-beta-D-GlcpNAc-(1->4)-D-GlcpNAc-yl group | Placed under new branch ‘amino oligosaccharide group’ |
| alpha-Neup5Ac-(2->6)-beta-D-Galp-(1->4)-beta-D-GlcpNAc-(1->2)-alpha-D-Manp-(1->3)-[alpha-Neup5-Ac-(2->6)-beta-D-Galp-(1->4)-beta-D-GlcpNAc-(1->2)-alpha-D-Manp-(1->6)]-beta-D-Manp-(1->4)-beta-D-GlcpNAc-(1->4)-[alpha-L-Fucp-(1->6)]-D-GlcpNAc-yl group | Placed under new branch ‘amino oligosaccharide group’ |
| alpha-Neup5Ac-(2->6)-beta-D-Galp-(1->4)-beta-D-GlcpNAc-(1->2)-alpha-D-Manp-(1->3)-[beta-D-Galp-(1->4)-beta-D-GlcpNAc-(1->2)-[beta-D-Galp-(1->4)-beta-D-GlcpNAc-(1->6)]-alpha-D-Manp-(1->6)]-beta-D-Manp-(1->4)-beta-D-GlcpNAc-(1->4)-D-GlcpNAc-yl group | Placed under new branch ‘amino oligosaccharide group’ |
| alpha-Neup5Ac-(2->6)-beta-D-Galp-(1->4)-beta-D-GlcpNAc-(1->2)-alpha-D-Manp-(1->3)-[beta-D-Galp-(1->4)-beta-D-GlcpNAc-(1->2)-alpha-D-Manp-(1->6)]-beta-D-Manp-(1->4)-beta-D-GlcpNAc-(1->4)-D-GlcpNAc-yl group | Placed under new branch ‘amino oligosaccharide group’ |
| beta-D-Galp-(1->3)-[alpha-L-Fucp-(1->4)]-beta-D-GlcpNAc-yl group | Placed under new branch ‘amino trisaccharide group’ |
| beta-D-Galp-(1->4)-beta-D-GlcpNAc-(1->2)-[beta-D-Galp-(1->4)-beta-D-GlcpNAc-(1->4)]-alpha-D-Manp-(1->3)-[beta-D-Galp-(1->4)-beta-D-GlcpNAc-(1->2)-alpha-D-Manp-(1->6)]-beta-D-Manp-(1->4)-beta-D-GlcpNAc-(1->4)-D-GlcpNAc-yl group | Placed under new branch ‘amino oligosaccharide group’ |
| beta-D-Galp-(1->4)-beta-D-GlcpNAc-(1->2)-alpha-D-Manp-(1->3)-beta-D-Manp-(1->4)-beta-D-GlcpNAc-(1->4)-D-GlcpNAc-yl group | Placed under new branch ‘amino hexasaccharide group’ |
| beta-D-galactosyl-(1->3)-N-acetyl-D-glucosaminyl group | Placed under new branch ‘amino disaccharide group’ |
| other glycosyl group | Removed |
| 2-O-methyl-4-(3-hydroxy-3-methylbutamido)-4,6-dideoxy-beta-D-glucosyl-(1->3)-alpha-L-rhamnosyl-(1->2)-alpha-L-rhamnosyl group | Placed under new branch ‘other amino trisaccharide group’ |
| 4-deoxy-beta-D-mannopyranosyl-(1->2)-beta-D-mannopyranosyl-(1->2)-beta-D-mannopyranosyl group | Placed under new branch ‘other glycosyl trisaccharide group’ |
| L-glycero-alpha-D-manno-heptosyl-(1->7)-L-glycero-alpha-D-manno-heptosyl group | Placed under new branch ‘other glycosyl disaccharide group’ |
| alpha-D-Galp-(1->2)-[alpha-D-Galp-(1->6)]-beta-D-Galp-yl group | Placed under new branch ‘other glycosyl trisaccharide group’ |
| alpha-D-Galp-(1->3)-beta-D-Galp-(1->4)-beta-D-Glcp-yl group | Placed under new branch ‘other glycosyl trisaccharide group’ |
| alpha-D-Rhap4NFo-(1->2)-alpha-D-Rhap4NFo(1->3)-alpha-D-Rhap4NFo-yl group | Placed under new branch ‘other amino trisaccharide group’ |
| alpha-D-Rhap4NFo-(1->2)-alpha-D-Rhap4NFo-(1->2)-alpha-D-Rhap4NFo-(1->2)-alpha-D-Rhap4NFo-(1->2)-alpha-D-Rhap4NFo-(1->2)-alpha-D-Rhap4NFo-yl group | Placed under new branch ‘amino hexasaccharide group’ |
| alpha-D-Rhap4NFo-(1->2)-alpha-D-Rhap4NFo-(1->2)-alpha-D-Rhap4NFo-(1->2)-alpha-D-Rhap4NFo-(1->2)-alpha-D-Rhap4NFo-yl group | Placed under new branch ‘amino pentasaccharide group’ |
| alpha-D-Rhap4NFo-(1->3)-alpha-D-Rhap4NFo-(1->2)-alpha-D-Rhap4NFo-(1->2)-alpha-D-Rhap4NFo-(1->2)-alpha-D-Rhap4NFo-yl group | Placed under new branch ‘amino pentasaccharide group’ |
| alpha-D-galactosyl-(1->2)-D-galactosyl group | Placed under new branch ‘other glycosyl disaccharide group’ |
| alpha-D-galactosyl-(1->2)-beta-D-galactosyl group | Placed under new branch ‘other glycosyl disaccharide group’ |
| alpha-D-galactosyl-(1->3)-D-galactosyl group | Placed under new branch ‘other glycosyl disaccharide group’ |
| alpha-D-galactosyl-(1->3)-alpha-D-galactosyl group | Placed under new branch ‘other glycosyl disaccharide group’ |
| alpha-D-galactosyl-(1->4)-beta-D-galactosyl group | Placed under new branch ‘other glycosyl disaccharide group’ |
| alpha-D-galactosyl-(1->6)-beta-D-galactosyl group | Placed under new branch ‘other glycosyl disaccharide group’ |
| alpha-L-Fucp-(1->2)-[alpha-D-Galp-(1->3)]-beta-D-Galp-(1->3)-alpha-D-GalpNAc-yl group | Placed under new branch ‘amino tetrasaccharide group’ |
| alpha-L-Fucp-(1->2)-[alpha-D-Galp-(1->3)]-beta-D-Galp-(1->3)-beta-D-GalpNAc-yl group | Placed under new branch ‘amino tetrasaccharide group’ |
| alpha-L-Fucp-(1->2)-[alpha-D-GalpNAc-(1->3)]-beta-D-Galp-(1->3)-alpha-D-GalpNAc-yl group | Placed under new branch ‘amino tetrasaccharide group’ |
| alpha-L-Fucp-(1->2)-[alpha-D-GalpNAc-(1->3)]-beta-D-Galp-(1->3)-beta-D-GalpNAc-yl group | Placed under new branch ‘amino tetrasaccharide group’ |
| alpha-L-Fucp-(1->2)-beta-D-Galp-(1->3)-alpha-D-GalpNAc-yl group | Placed under new branch ‘other amino trisaccharide group’ |
| alpha-L-Fucp-(1->2)-beta-D-Galp-(1->3)-beta-D-GalpNAc-yl group | Placed under new branch ‘other amino trisaccharide group’ |
| alpha-L-fucosyl group | Placed under new branch ‘other glycosyl monosaccharide group’ |
| alpha-L-fucosyl-(1->2)-beta-D-galactosyl group | Placed under new branch ‘other glycosyl disaccharide group’ |
| alpha-Neup5Ac-(2->8)-alpha-Neup5Ac-(2->8)-alpha-Neup5Ac-yl group | Placed under new branch ‘other amino trisaccharide group’ |
| alpha-Neup5Gc-(2->3)-beta-D-Galp-(1->4)-D-Glcp-yl group | Placed under new branch ‘other amino trisaccharide group’ |
| beta-D-Araf-(1->2)-alpha-D-Araf-(1->5)-[beta-D-Araf-(1->2)-alpha-D-Araf-(1->3)]-alpha-D-Araf-(1->5)-alpha-D-Araf-yl group | Placed under new branch ‘other glycosyl hexasaccharide group’ |
| beta-D-galactofuranosyl group | Placed under new branch ‘other glycosyl monosaccharide group’ |
| beta-D-mannopyranosyl-(1->2)-beta-D-mannopyranosyl group | Placed under new branch ‘other glycosyl disaccharide group’ |
| beta-D-mannopyranosyl-(1->2)-beta-D-mannopyranosyl-(1->2)-4-deoxy-beta-D-mannopyranosyl group | Placed under new branch ‘other amino trisaccharide group’ |
| beta-D-mannopyranosyl-(1->2)-beta-D-mannopyranosyl-(1->2)-beta-D-mannopyranosyl group | Placed under new branch ‘other amino trisaccharide group’ |
| beta-D-mannosyl-(1->2)-beta-D-mannosyl-(1->2)-beta-D-mannosyl-(1->2)-beta-D-mannosyl group | Placed under new branch ‘other amino tetrasaccharide group’ |
| beta-D-xylosyl group | Placed under new branch ‘other glycosyl monosaccharide group’ |
| organyl group | Removed |
| other organyl group | Removed |
| 2,4,6-trinitrophenyl group | Placed under new branch ‘nitrophenyl group’ |
| 2,4-dinitrophenyl group | Placed under new branch ‘nitrophenyl group’ |
| 2,6-dinitrophenyl group | Placed under new branch ‘nitrophenyl group’ |
| 2-carboxy-4,6-dinitrophenyl group | Placed under new branch ‘nitrophenyl group’ |
| allylmercaptomethylpenicilloyl group | Placed under ‘penicilloyl group’ |
| alpha-D-Rhap4NFo-(1->2)-alpha-D-Rhap4NFo-(1->2)-alpha-D-Rhap4NFo-(1->2)-alpha-D-Rhap4NFo-(1->2)-alpha-D-Rhap4NFo-(1->2)-alpha-D-Rhap4NFoO[CH2]5C(O)NH2CH2CH2NH2-3,4-dioxocyclobutylaminomethyl group | Placed under new branch ‘glycolipid group’ |
| alpha-D-Rhap4NFo-(1->2)-alpha-D-Rhap4NFo-(1->3)-alpha-D-Rhap4NFo-(1->2)-alpha-D-Rhap4NFo-(1->2)-alpha-D-Rhap4NFo-(1->2)-alpha-D-Rhap4NFoO[CH2]5C(O)NH2CH2CH2NH2-3,4-dioxocyclobutylaminomethyl group | Placed under new branch ‘glycolipid group’ |
| alpha-D-Rhap4NFo-(1->2)-alpha-D-Rhap4NFo-(1->3)-alpha-D-Rhap4NFo-(1->2)-alpha-D-Rhap4NFoO[CH2]5C(O)NH2CH2CH2NH2-3,4-dioxocyclobutylaminomethyl group | Placed under new branch ‘glycolipid group’ |
| alpha-D-Rhap4NFo-(1->2)-alpha-D-Rhap4NFo-(1->3)-alpha-D-Rhap4NFoO[CH2]5C(O)NH2CH2CH2NH2-3,4-dioxocyclobutylaminomethyl group | Placed under new branch ‘glycolipid group’ |
| alpha-D-Rhap4NFo-(1->3)-alpha-D-Rhap4NFo-(1->2)-alpha-D-Rhap4NFoO[CH2]5C(O)NH2CH2CH2NH2-3,4-dioxocyclobutylaminomethyl group | Placed under new branch ‘glycolipid group’ |
| alpha-D-Rhap4NFo-(1->3)-alpha-D-Rhap4NFoO[CH2]5C(O)NH2CH2CH2NH2-3,4-dioxocyclobutylaminomethyl group | Placed under new branch ‘glycolipid group’ |
| carbenicilloyl group | Placed under ‘penicilloyl group’ |
| dimethoxyphenylpenicilloyl group | Placed under ‘penicilloyl group’ |
| o-nitrophenyl group | Placed under new branch ‘nitrophenyl group’ |
| p-nitrophenyl group | Placed under new branch ‘nitrophenyl group’ |
| trimellityl group | Placed under ‘other carboxylic acid’ |
| penicilloyl group | Placed under ‘penicillin’ |
| 6-formamidopenicilloyl group | Remaining a child of ‘penicilloyl group’ |
| amoxicillanyl group | Remaining a child of ‘penicilloyl group’ |
| amoxicilloyl group | Remaining a child of ‘penicilloyl group’ |
| ampicillanyl group | Remaining a child of ‘penicilloyl group’ |
| ampicilloyl group | Remaining a child of ‘penicilloyl group’ |
| benzylpenicillanyl group | Remaining a child of ‘penicilloyl group’ |
| benzylpenicilloyl group | Remaining a child of ‘penicilloyl group’ |
| flucloxacilloyl group | Remaining a child of ‘penicilloyl group’ |
| flucloxcillanyl group | Remaining a child of ‘penicilloyl group’ |
| phenethicilloyl group | Remaining a child of ‘penicilloyl group’ |
| phenoxymethylpenicillanyl group | Remaining a child of ‘penicilloyl group’ |
| phenoxymethylpenicilloyl group | Remaining a child of ‘penicilloyl group’ |
| sulbenicilloyl group | Remaining a child of ‘penicilloyl group’ |
| other organic group | Removed |
| ->3)-beta-D-Galp-(1->3)-beta-D-GalpNAc-(1->4)-[alpha-Neup5Ac-(2->8)-alpha-Neup5Ac-(2->8)-alpha-Neup5Ac-(2->3)]-beta-D-Galp-yl group | Placed under new branch ‘amino hexasaccharide group’ |
| 4-arsonophenyldiazenyl group | Placed under new branch ‘nitrophenyl group’ |
| 9-(2-O-methyl-alpha-L-rhamnosyloxy)nonanoylamino group | Placed under new branch ‘glycolipid group’ |
| N(4)-[alpha-L-fucosyl-(1->3)-N-acetyl-4-O-glycosyl-D-glucosaminyl]-L-asparagine residue | Placed under ‘asparagine and asparagine derivative’ |
| N(4)-{alpha-D-Manp-(1->2)-alpha-D-Manp-(1->2)-alpha-D-Manp-(1->3)-[alpha-D-Manp-(1->3)-[alpha-D-Manp-(1->2)-alpha-D-Manp-(1->6)]-alpha-D-Manp-(1->6)]-beta-D-Manp-(1->4)-beta-D-GlcpNAc-(1->4)-beta-D-GlcpNAc}-Asn residue | Placed under new branch ‘amino oligosaccharide group’ |
| N(4)-{alpha-Neu5Ac-(2->6)-beta-D-Gal-(1->4)-beta-D-GlcNAc-(1->2)-alpha-D-Man-(1->3)-[alpha-Neu5Ac-(2->6)-beta-D-Gal-(1->4)-beta-D-GlcNAc-(1->2)-alpha-D-Man-(1->6)]-beta-D-Man-(1->4)-beta-D-GlcNAc-(1->4)-[alpha-L-Fuc-(1->6)]-D-GlcNAc}-L-Asn residue | Placed under new branch ‘amino oligosaccharide group’ |
| N(4)-{alpha-Neu5Ac-(2->6)-beta-D-Gal-(1->4)-beta-D-GlcNAc-(1->2)-alpha-D-Man-(1->3)-[alpha-Neu5Ac-(2->6)-beta-D-Gal-(1->4)-beta-D-GlcNAc-(1->2)-alpha-D-Man-(1->6)]-beta-D-Man-(1->4)-beta-D-GlcNAc-(1->4)-beta-D-GlcNAc}-L-Asn residue | Placed under new branch ‘amino oligosaccharide group’ |
| N(6)-(glycylglycyl)-L-lysine residue | Placed under ‘L-lysine and L-lysine derivative’ |
| N(6)-[H2N-NLFQVVHNSYNRPAYSPG-N-{54-amino-7,10,18,21,29,32,40,43,51,54-decaoxo-3,14,25,36,47-pentakis-[5-({2-[alpha-L-Rhap-(1->3)-beta-D-GlcpO]ethyl}sulfinyl)pentanoyl]-3,6,11,14,17,22,25,28,33,36,39,44,47,50-tetradecaazatetrapentacont-1-yl}]adipamido group | Placed under ‘other organic molecular entity’ |
| N-acetyl-alpha-D-galactosaminyl-L-serine residue | Placed under ‘serine and serine derivative’ |
| N-{alpha-Man-(1->3)-[alpha-Man-(1->3)-[alpha-Man-(1->6)]-alpha-Man-(1->6)]-beta-Man-(1->4)-beta-GlcNAc-(1->4)-beta-GlcNac}-L-Asn residue | Placed under new branch ‘amino heptasaccharide group’ |
| O-(N-acetyl-alpha-D-galactosaminyl)-L-threonino group | Placed under ‘threonine and threonine derivative’ |
| O-[N-acetyl-alpha-neuraminyl-(2->6)-N-acetyl-alpha-D-galactosaminyl]-L-serine residue | Placed under ‘L-serine and L-serine derivative’ |
| O-[N-acetyl-alpha-neuraminyl-(2->6)-N-acetyl-alpha-D-galactosaminyl]-L-threonine residue | Placed under ‘threonine and threonine derivative’ |
| [2)-alpha-L-Rhap3/4Ac-(1->] residue | Placed under new branch ‘other monosaccharide derivative group’ |
| [4)-alpha-D-Glcp-(1->4)-alpha-D-Glcp-(1->4)-alpha-D-Glcp-(1->] residue | Placed under new branch ‘other trisaccharide derivative group’ |
| phosphocholine group | Placed under ‘other organonitrogen molecular entity’ |
| phthaloyl group | Placed under new branch ‘divalent carboacyl group’ |
| succinyl group | Placed under new branch ‘divalent carboacyl group’ |
| tyrosine residue | Placed under ‘tyrosine and tyrosine derivative’ |
| phenylazo groups | Placed under ‘benzene molecular entity’ |
| (2-carboxylatophenyl)azo group | Remaining a child of ‘phenylazo groups’ |
| (2-nitrophenyl)azo group | Remaining a child of ‘phenylazo groups’ |
| (2-sulfonatophenyl)azo group | Remaining a child of ‘phenylazo groups’ |
| (3-carboxylatophenyl)azo group | Remaining a child of ‘phenylazo groups’ |
| (3-nitrophenyl)azo group | Remaining a child of ‘phenylazo groups’ |
| (3-sulfonatophenyl)azo group | Remaining a child of ‘phenylazo groups’ |
| (4-carboxylatophenyl)azo group | Remaining a child of ‘phenylazo groups’ |
| (4-nitrophenyl)azo group | Remaining a child of ‘phenylazo groups’ |
| (4-sulfonatophenyl)azo group | Remaining a child of ‘phenylazo groups’ |
| 3-(leucinocarbonyl)phenylazo group | Remaining a child of ‘phenylazo groups’ |
| [2-(hydroxyarsinato)phenyl]azo group | Remaining a child of ‘phenylazo groups’ |
| [4-(hydroxyarsinato)phenyl]azo group | Remaining a child of ‘phenylazo groups’ |
| phenylazo group | Remaining a child of ‘phenylazo groups’ |
| univalent carboacyl group | Placed under ‘carboxylic acid’ |
| (3-bromo-4-hydroxy-5-nitrophenyl)acetyl group | Placed under new branch ‘nitrophenylacetyl group’ and remaining a child of ‘univalent carboacyl group’ |
| (4-hydroxy-3-iodo-5-nitrophenyl)acetyl group | Placed under new branch ‘nitrophenylacetyl group’ and remaining a child of ‘univalent carboacyl group’ |
| (4-hydroxy-3-nitrophenyl)acetyl group | Placed under new branch ‘nitrophenylacetyl group’ and remaining a child of ‘univalent carboacyl group’ |
| (Z)-3-carboxyprop-2-enoyl group | Remaining a child of ‘univalent carboacyl group’ |
| 2-chlorobenzoyl group | Placed under ‘monochlorobenzene’ and remaining a child of ‘univalent carboacyl group’ |
| 4-(alpha-L-rhamnosyloxy)butanoyl group | Placed under new branch ‘other monosaccharide derivative group’ and remaining a child of ‘univalent carboacyl group’ |
| 4-chlorobenzoyl group | Placed under ‘monochlorobenzene’ and remaining a child of ‘univalent carboacyl group’ |
| acetyl group | Remaining a child of ‘univalent carboacyl group’ |
| amoxicilloyl group | Placed under ‘penicilloyl group’ and remaining a child of ‘univalent carboacyl group’ |
| ampicilloyl group | Placed under ‘penicilloyl group’ and remaining a child of ‘univalent carboacyl group’ |
| aztreonyl group | Placed under ‘penicilloyl group’ and remaining a child of ‘univalent carboacyl group’ |
| benzylpenicilloyl group | Placed under ‘penicilloyl group’ and remaining a child of ‘univalent carboacyl group’ |
| carbamoyl group | Remaining a child of ‘univalent carboacyl group’ |
| lipoyl group | Remaining a child of ‘univalent carboacyl group’ |
| p-nitrobenzoyl group | Placed under new branch ‘nitrophenyl group’ and remaining a child of ‘univalent carboacyl group’ |
| phenoxymethylpenicilloyl group | Placed under ‘penicilloyl group’ and remaining a child of ‘univalent carboacyl group’ |
| phthalyl group | Placed under ‘other benzene’ and remaining a child of ‘univalent carboacyl group’ |
| purin-6-oyl group | Placed under ‘purine’ and remaining a child of ‘univalent carboacyl group’ |
| trifluoroacetyl group | Remaining a child of ‘univalent carboacyl group’ |
| other group | Removed |
| dansyl group | Placed under ‘other benzene’ and under new branch ‘sulfonyl group’ |
| tosyl group | Placed under ‘other benzene’ and under new branch ‘sulfonyl group’ |
