# Supplementary material for: An immunologically friendly classification of non-peptidic ligands
Source: Database (Oxford). 2021 Mar 27;2021:baab014. doi: 10.1093/database/baab014 (PMC8001080; doi:10.1093/database/baab014)
Supplement: baab014_Supp [file baab014_supp.zip › Non-peptidic Manuscript - Supplemental Table 2 (Resubmission).docx]

**Supplemental Table 2.** The user test that was conducted, comprised of 20 commonly studied non-peptidic epitopes separated into 2 test sets. Three participants were asked to find test set 1 in the new tree, and test set 2 in the old tree, whilst the other three participants were asked to complete the inverse.

| **Test Set 1** | **Test Set 2** |
| --- | --- |
| 1-O-(alpha-D-galactosyl)-N-hexacosanoylphytosphingosine | 2,4-dinitrophenyl group |
| phosphatidyl-L-serine | beta-D-Gal-(1->3)-beta-D-GalNAc-(1->4)-[alpha-Neu5Ac-(2->8)-alpha-Neu5Ac-(2->3)]-beta-D-Gal-(1->4)-beta-D-Glc-(1<->1')-Cer |
| alpha-D-galactosyl-(1->3)-D-galactose | phosphatidylethanolamine |
| benzylpenicillin | 4-(ethoxymethylene)-2-phenyloxazol-5-one |
| phosphocholine | phosphatidylinositol |
| phosphatidic acid | nickel atom |
| alpha-L-Fucp-(1->3)-[beta-D-Galp-(1->4)]-beta-D-GlcpNAc | trimellitic anhydride |
| sulfamethoxazole | 1,4-phenylenediamine |
| 3-deoxy-alpha-D-manno-oct-2-ulopyranosonic acid | ganglioside GM1 |
| fluorescein isothiocyanate | (4-hydroxy-3-nitrophenyl)acetyl group |
