# Supplementary figures and images for: An immunologically friendly classification of non-peptidic ligands
Source: Database (Oxford). 2021 Mar 27;2021:baab014. doi: 10.1093/database/baab014 (PMC8001080; doi:10.1093/database/baab014)

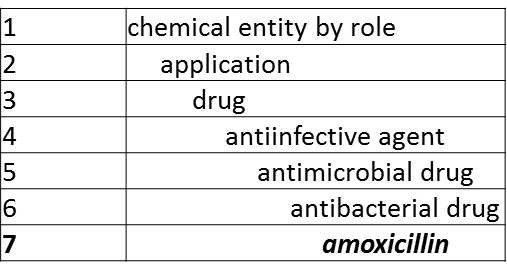


**Supplemental Figure 2a.** The location of amoxicillin in the chemical entity by role tree.

Supplement: baab014_Supp [file baab014_supp.zip › Non-peptidic Manuscript - Supplemental Figure 2a (Resubmission).docx]

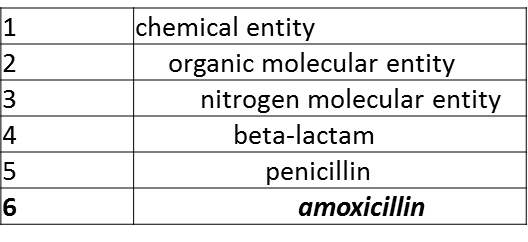


**Supplemental Figure 2b.** The location of amoxicillin in the structural chemical entity tree.

Supplement: baab014_Supp [file baab014_supp.zip › Non-peptidic Manuscript - Supplemental Figure 2b (Resubmission).docx]
